# Supplementary material for: Crystalline polymeric carbon dioxide stable at megabar pressures
Source: Nat Commun. 2018 Aug 8;9:3148. doi: 10.1038/s41467-018-05593-8 (PMC6082874; doi:10.1038/s41467-018-05593-8)
Supplement: Supplementary file 1 — Supplementary Information [file 41467_2018_5593_MOESM1_ESM.pdf]

SUPPLEMENTARY INFORMATION

Dziubek et al.

Crystalline polymeric carbon dioxide stable at megabar pressures

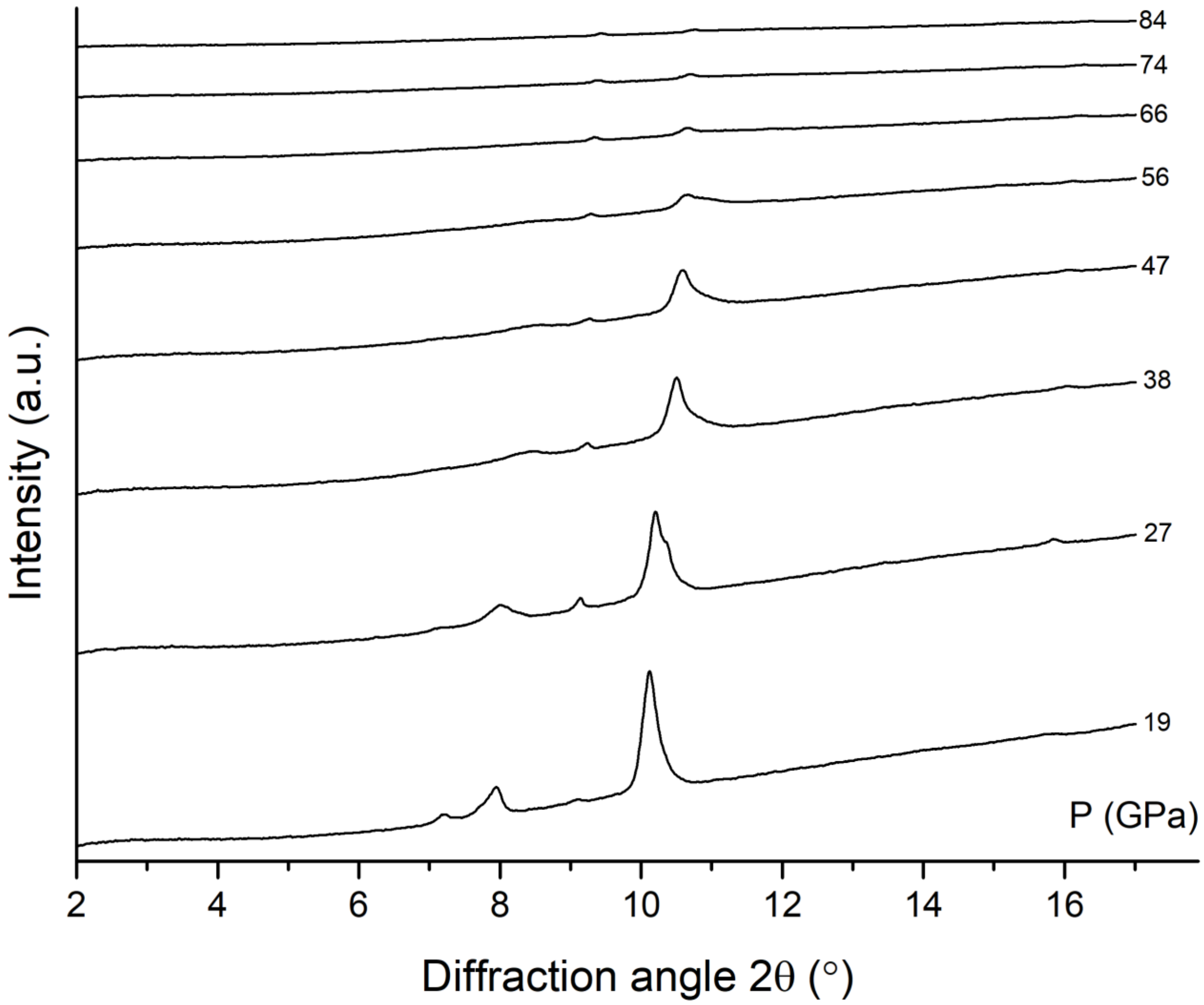

Supplementary Figure 1 XRD patterns of CO_2 observed on compression at ambient temperature. The beam was focused in the center of the sample where only pure CO_2 was found, however additional peaks of the Re gasket become visible as the dimensions of the pressure chamber changed with pressure.

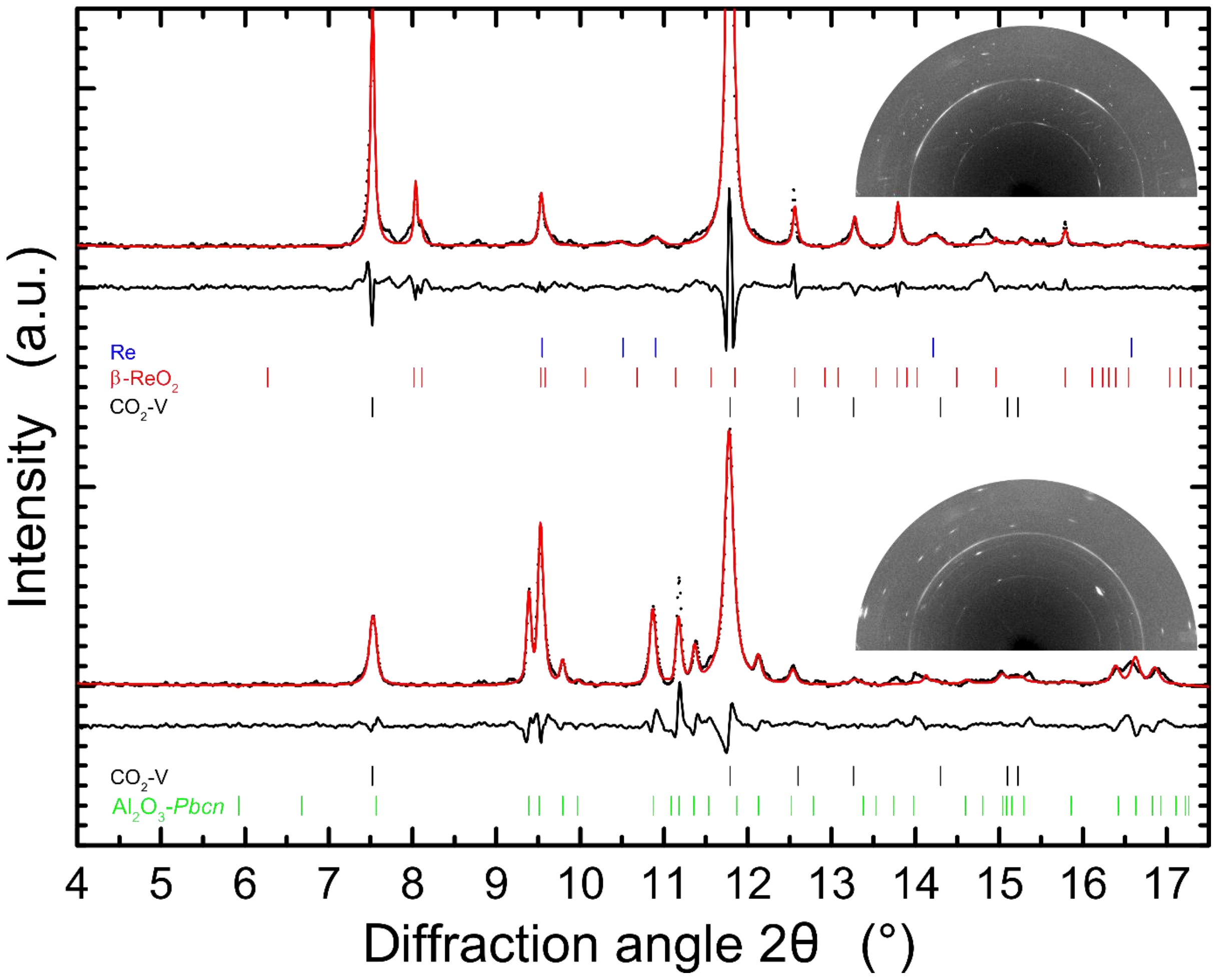

Supplementary Figure 2 Characterizing lateral regions of the sample. Integrated XRD patterns measured at two different sample points after the second laser heating cycle (T = 295 K, P ≈ 108 GPa). 2D panoramic oscillation images (φ = ±7°) corresponding to the reported patterns are shown as insets. Black dots are experimental data, red line is the fit, and the black line represents the difference between the two. The phase assignments are shown as tick marks: black for the reflections of CO_2-V, red for *β*-ReO_2, blue for Re and green for the high-pressure high-temperature phase of Al_2O_3 [of Rh_2O_3 (II) structure, space group *Pbcn*]. Rietveld method using spherical harmonics for preferred orientation was used for fitting the reflections of CO_2-V and Al_2O_3-*Pbcn*, while for fitting the peaks of Re and *β*-ReO_2 LeBail method was applied. The refined lattice parameters, *a* = 4.48, *b* = 5.29, *c* = 4.27 for *β*-ReO_2 and *a* = 6.40, *b* = 4.38, *c* = 4.56 for Al_2O_3-*Pbcn* agree reasonably well with the values estimated on the basis of thermodynamic conditions and equations of state[1,2].

References

1. Lin, J.-F. et al. Crystal structure of a high-pressure/high-temperature phase of alumina by in situ X-ray diffraction. *Nature Materials* **3**, 389-393 (2004).

2. Santamaría-Pérez, D. et al. Exploring the chemical reactivity between carbon dioxide and three transition metals (Au, Pt, and Re) at high-pressure, high-temperature conditions. *Inorg. Chem.* **55**, 10793-10799 (2016).
